# Some New or Imperfectly Understood Points in the Anatomy of the Horse’s Foot*

**Published:** 1880-01

**Authors:** John A. McLaughlin

**Affiliations:** Student Veterinary Medicine


### Art. V.—SOME NEW OR IMPERFECTLY UNDERSTOOD POINTS IN THE ANATOMY OF THE HORSE'S FOOT.*

By JOHN A. McLAUGHLIN, Jr.,

Student Veterinary Medicine.

(*From the Laboratory of Comparative Anatomy and Pathology, Columbia Veterinary College, II*).

### Introductory Remarks.

IT is no exaggeration to state that over one-half of the equine disorders which the veterinarian is called upon to treat are located in or have taken their origin from the hoof, and the tissues enclosed within its horny case.

Comparatively insignificant in the multidigitated mammals, the ungual structures in Solipeds attain an importance, with which their intricate vascular and nervous supply is in full accord.

The student of Veterinary Medicine is often embarrassed by reading or hearing of contradictory views in regard to the pathology and treatement of the diseases of this important apparatus. *A priori* one should suppose that a reference to the anatomical structures and physiology of these structures in the foot would render all these doubtful points more certain, and would give a far safer basis for pathology and treatment than mere abstractions based on hap-hazard experiment, or even of routine experience.

This consideration has induced me to conduct special researches on the topographical anatomy, the histology, and the mechanism of the healthy foot. Although we have the excellent manual of Leisering, and the able articles of Fleming to guide us in this interesting and practical field, yet it must be confessed that in many respects still greater clearness is desirable.

* Lack of space in the present number forbids our giving this article in full.

Thus to mention but a single instance in point. Fleming insists that the podophyllous tissue, although it secretes horn in laminitis, does not do so under normal circumstances. My results so far are quite contradictory to those of the great British authority, and the matter will be fully discussed in these pages.

Then again, although the structures of and around the foot articulation have been repeatedly studied, no one has, in accessible publications, given a clear topographical description of the relations of this joint and its bursa.

I have undertaken in the limits of this article to discuss the following points. If I have in the course of my descriptions repeated many well-known and long-established statements, the reader will recollect that such repetition was necessary to the unity of the subject:

1. The coarse descriptive anatomy of the foot structures.
2. The normal growth of the hoof-horn.
3. The growth of the hoof-horn in the fœtus.
4. The changes of the coarse structures with age.
5. The topographical anatomy of the hoof in mathematical projection.
6. The mechanism of the foot.
7. The vascular supply and innervation ot the foot.

(*To be continued.*)

